# Real-Time Modeling Should Be Routinely Integrated into Outbreak Response

**DOI:** 10.4269/ajtmh.18-0150

**Published:** 2018-04-02

**Authors:** Daniel G. Bausch, John Edmunds

**Affiliations:** 1 UK Public Health Rapid Support Team, London, United Kingdom;; 2 London School of Hygiene and Tropical Medicine, London, United Kingdom;; 3 Public Health England, London, United Kingdom

Outbreaks may start with a single infection, but where they go from there ranges from a few sporadic cases of no major global consequence to a full-scale pandemic. Predicting how big or small an outbreak will be has obvious use in mounting the appropriate public health response; “getting it right” allows national and international responders to gauge the human and material resources necessary, preparing and mobilizing commensurately to stem transmission and save lives. Getting it wrong risks not only severe consequences in human suffering but also reputational consequences; whether justified or not, the World Health Organization (WHO) was heavily criticized for both overreacting to the 2009 H1N1 influenza virus pandemic and dragging its feet in recognizing the magnitude and severity of the 2013–2016 outbreak of Ebola virus disease in West Africa.^1^

Mathematical modeling of disease has traditionally been an academic and post hoc discipline, analyzing data after events occur to inform on possible future transmission. However, with improvements in the speed and quality of data collection and modern statistical (often Bayesian) methods, it is becoming increasingly feasible to conduct real-time quantitative situational assessments and, thereby, forecast the likely course of an ongoing epidemic.^2^ In this month’s *American Journal of Tropical Medicine and Hygiene*, Graham et al.^3^ report on their attempts at real-time modeling of a measles outbreak in Guinea, West Africa.Different model variants produced different forecasts. Although all considerably overestimated the number of future cases, the models nevertheless correctly forecasted the overall trajectory of the outbreak, information that was rapidly communicated to WHO. Interestingly, using corrected data sets that the authors felt were most reliable brought predictions more in line with observation for some areas of the outbreak, but less in line for others.

Equally important to their modeling results are Graham et al.’s perspectives on the challenges of real-time outbreak modeling. Number one on the list of challenges is the quality of data available; in the setting where outbreaks most often occur—remote areas of low- and middle-income countries (LMICs) with weak or failed health and surveillance systems—high-quality data are unlikely to be available, at least in the early stages. In addition, accurate predictions typically face the challenges of the rapid evolution of outbreaks, in which a complex host of direct (e.g., enhanced surveillance and case finding, vaccination campaigns) and indirect (e.g., successful messaging to the community resulting in behavioral change) factors and interventions must be considered.

Despite these challenges, the potential gains from real-time outbreak modeling are far-reaching.^4,5^ In addition to the most obvious contribution, forecasting attack rates and incidence, modeling can shed light on the underlying factors that may have led to and continue to sustain the outbreak, including population immunity and susceptibility, transmissibility, the effectiveness of interventions, and seasonality. Even models that lack precision, as was the case in predicting the measles outbreak in Guinea, may be useful if the overall trajectory and order of magnitude of an outbreak can be forecasted with reasonable confidence. Furthermore, modeling itself may serve as a motivating action, leading to enhanced interventions and better outbreak control.^4^ While working to refine models to improve precision, Graham et al.’s simple forecast classifications of “pessimistic,” “mid,” or “optimistic” may serve as a readily comprehensible and actionable categorization.

Although requests for modeling and analyses are becoming more frequent, they often come through networks of informal contacts on an ad hoc basis and are typically under-resourced, limiting the ability to respond adequately to the increasing demand for these services. Furthermore, the data on which the models are built are usually made available on a confidential basis, restricting the audience for the modeling analyses and prohibiting or slowing the sharing of results with other responding organizations. It is time to include real-time applied modeling as a regular pillar of outbreak response, not because it is yet perfect, but because of the potential it holds, and because the experience gained through regular application will certainly help to improve precision. However, to derive the most from modeling, capacities must be created and teams prepared in advance.

In 2016 the United Kingdom created the UK Public Health Rapid Support Team (UK-PHRST), a joint effort between Public Health England and the London School of Hygiene and Tropical Medicine (LSHTM), with a mandate to respond to outbreaks, conduct innovative research to generate evidence on best practices for outbreak control, and build capacity for outbreak response in LMICs.^6^ The UK-PHRST comprises a multidisciplined team of epidemiologists, laboratory microbiologists, clinical researchers, infection prevention and control experts, social scientists, data scientists, and logisticians ready and resourced for deployment to outbreaks in LMICs within 48 hours’ notice. To date, the UK-PHRST has responded to outbreaks in Ethiopia, Nigeria, Sierra Leone, Madagascar, and Bangladesh.

The LSHTM and UK-PHRST are now taking steps to incorporate real-time mathematical modeling and outbreak analysis as a fundamental component of the epidemic response. Recent examples include modeling of a diphtheria outbreak in a forcibly displaced Rohingya population in Bangladesh and assessing oral cholera vaccination options during the 2017 cholera epidemic in Yemen. Having an integrated and resourced modeling component will allow the UK-PHRST to develop and vet various modeling approaches and plan responses in advance of outbreaks and to work out many of the logistical and operational challenges faced by teams, however skilled, thrown together in an emergency.

The UK-PHRST provides an important opportunity to integrate skilled modelers into the field team during outbreaks to collect high-quality data that can be rapidly fed into models and analyzed for real-time forecasting. Real-time feedback on data gaps and modeling misspecifications will allow for rapid corrections and revised forecasts. Furthermore, the UK-PHRST plans to explore the integration of modern laboratory (e.g., real-time pathogen sequencing in the field made available through mobile sequencing platforms such as the Oxford Nanopore MinION system) and surveillance (e.g., outbreak tracking using “big data,” such as short message system texting) tools into the analyses, with the potential to bring about a rapid and profound understanding of the present and future direction of an outbreak, facilitating tailoring of the response accordingly.

Many challenges exist to reap the full benefits of real-time outbreak modeling, including developing guidelines, agreeing on parameters for rapid data sharing while ensuring confidentiality of patients and other sensitive data, and technical challenges with regard to bioinformatics. Routinely incorporating real-time mathematical modeling into the traditional pillars of outbreak response (e.g., case finding, contact tracing, laboratory diagnosis, case management, community outreach, and messaging), with appropriate advance planning and adequate resources, will help push this valuable contribution to outbreak response forward, with the potential for increasing precision in forecasts, optimal planning and use of resources, and lives saved.
